# Theoretical and Experimental Studies of Hydrogen Bonded Dihydroxybenzene Isomers Polyurethane Adhesive Material

**DOI:** 10.3390/polym14091701

**Published:** 2022-04-21

**Authors:** Joyanta K. Saha, Mohammad Mizanur Rahman, Md Bashirul Haq, Dhafer A. Al Shehri, Joonkyung Jang

**Affiliations:** 1 Department of Chemistry, Jagannath University, Dhaka 1100, Bangladesh; 2 Interdisciplinary Research Center for Advanced Materials, King Fahd University of Petroleum & Minerals, Dhahran 31261, Saudi Arabia; 3 Department of Petroleum Engineering, College of Petroleum and Geosciences, King Fahd University of Petroleum & Minerals, Dhahran 31261, Saudi Arabia; bhaq@kfupm.edu.sa (M.B.H.); alshehrida@kfupm.edu.sa (D.A.A.S.); 4 Department of Nanoenergy Engineering, Pusan National University, Busan 46241, Korea; jkjang@pusan.ac.kr

**Keywords:** polyurethane, hydrogen bond, DFT, catechol, hydroquinone

## Abstract

Hydrogen bonding in polyurethane (PU) is imposed by molecular parameters. In this study, the effect of structural isomerism of certain monomers on hydrogen bonding of waterborne polyurethane (WBPU) was studied theoretically and experimentally. Two dihydroxybenzene (DHB)-based structural isomers such as catechol (CC) and hydroquinone (HQ), with different OH positions on the inner benzene core, had been used. Two series of WBPU dispersions were prepared using CC and HQ with defined contents. The binding energies between the catechol (CC)/hydroquinone (HQ) (respective OH group) and urethane/urea were calculated theoretically. By using a density functional theory (DFT) method, it was found that the largest binding energy between the urea and CC was higher than that of urea and HQ. The FT-IR analysis of synthesized polymer was also carried out to compare the results with the theoretical values. The CC-based polymers showed a stronger hydrogen bond both theoretically and experimentally than those for HQ-based polymers. The higher level of hydrogen bond was reflected in their properties of CC-based polymers. The adhesive strength, thermal stability, and hydrophobicity were higher for CC-based materials than those for HQ-based materials. The adhesive strength was increased 25% with the addition of 2.0 wt% CC content. This adhesive strength slightly deviated at a moderately high temperature of 80 °C.

## 1. Introduction

The theoretical calculation of bonding energy in the polymer is important. It helps to screen the formulations to avoid many unnecessary lab reactions; eventually, it can save the chemicals and time. Thus, it is highly beneficial for the environment by using only necessary chemicals and is cost-effective by arranging the required chemicals only. The widely used theoretical calculation method of bonding is the density functional theory (DFT) method. Especially, the hydrogen bond energy calculation by the DFT method has been proved to be an efficient tool. The value of binding energy is an indication of the strength of hydrogen bond of functional groups. The binding energy value changes with the nature and content of the monomers. A higher value indicates more hydrogen bonding between certain functional groups in the polymer. This will ultimately improve the properties. Thus, this calculation will help to choose the appropriate monomers and their contents to synthesize polymers in a laboratory [1,2,3,4].

Polyurethane (PU) is a multifunctional polymer. Compared to other traditional polymers, PU usually possesses superior properties. Nowadays, PU is widely being used in many different areas especially in coating, adhesive, and biomaterials [5,6,7,8]. By adopting new techniques, the PU material is extended its new areas of applications, especially for harsh conditions. Urethane (NHCOO) and urea (NHCONH) are two main functional groups in PU. The urethane group is formed by reacting hydroxyl (OH) and isocyanate (NCO) groups; and the urea group by reacting amine (NH_2_) and NCO groups. Different polyether polyol, polyester polyol, acrylate polyol, and siloxane polyol with their different molecular weight acts for -OH group, whereas aliphatic and aromatic isocyanates act for the NCO group. These monomers not only form the urethane-urea groups but also contribute to hydrogen bond (HB) formation, providing their available electronegative atoms to participants. The newly formed urethane and urea groups also participate in hydrogen bonding. In our previous study [9], it had been shown that the hydrogen bond density, which is an indication of the strength of HB, was changed by changing monomers and their contents. The HB quantity increased up to a certain EDA content [9]. In another study, we showed that the HB increased with increasing the DMPA content [10]. We also showed that the HB increased when lignin was used along with EDA as a chain extender [11]. It had been shown that the polymer properties were hugely affected by their HB. The adhesive strength, hydrophobicity, mechanical strength, and corrosion resistance all increased with increasing HB [8,9,10,11]. Self-healing of materials was also improved by increasing HB [12]. Rahman et al. [12] used a certain level of monomer to have a supramolecular structure to increase the HB, which ultimately improved the self-healing nature of materials. However, excessive HB can derail the properties [9]. It is important to maintain the proper balance of monomers to keep the HB at a certain level.

PU is widely being used for adhesion purposes in the textile industry [13]. Unfortunately, commercial PU adhesives mostly contain toxic organic solvents, which are emitted during the synthesis and drying stages [5]. Thus, it threatens the environment as well as human health. Many countries restricted these organic solvents, used to save human health and the environment. Due to this limitation, the trends of using organic coatings slowly changed from solvent-based to water-based/waterborne polyurethane (WBPU) adhesives. Unfortunately, the water-based PU adhesive has comparatively less adhesive strength. Significant efforts were found by many researchers to improve the adhesive strength of WBPU. They used different DMPA contents [10], polyols [14], hardeners [10], crosslinkers [15] and nanoparticles [16]. The adhesive strength also varied with pressed temperature during the bonding of two nylon fabrics [10]. The adhesive strength was improved by maintaining a proper stoichiometric ratio of monomers [10]. The main challenge appears at moderately high temperatures and under water conditions. The adhesive strength usually falls in both cases [10,15]. Scientists are still working to improve the WBPU adhesion at moderately high temperatures and wet conditions.

There are monomers which are a mixture of isomers. The effect of isomers on properties is mostly overlooked in WBPU materials. The widely used monomer H_12_MDI is a mixture of isomers (cis-cis, trans-trans, cis-trans). Saralegi et al. [16] separated the isomers of H_12_MDI. The mechanical strength and thermal stability are hugely affected due to their different isomer structures. Catechol (CC) and hydroquinone (HQ) are isomers, with the same chemical formula as C_6_H_4_(OH)_2_ and a molecular weight of 110.1 g/mol, of 1, 2-dihydroxybenzene (DHB) and 1, 4-dihydroxybenzene, respectively. The DHB is used in polymeric materials to improve their mechanical strength, thermal stability, and hydrophobicity. The DHB is also used in PU. Ren et al. [17] used a CC-based compound in PU. The highest level of thermal stability, mechanical strength, and adhesive strength were recorded at the ratio 25:4 of NCO and CC. They proved that the proper CC content can only improve the properties at a significant level. Huang et al. [18] used HQ in PU to improve the mechanical and thermal properties. It is hard to find any report comparing the DHB isomer effect on WBPU properties. This scenario makes us interested in investigating the effect of hydrogen bonds (due to the different positions of -OH groups) between CC/HQ and urethane/urea groups on the properties. Such an investigation can further help to assess the effect of similar types of other isomeric monomer structures on polymer properties. We counted both theoretically and experimentally hydrogen bonds and their ultimate effect on WBPU properties. To the best of our knowledge, such a type of comparisons has not been considered yet, especially in PU materials. In this study, two series of WBPU dispersions, using defined CC and HQ contents, were prepared. The dispersion sacrificed their shelf life when the CC and HQ contents were above 2.0 wt%. Thus, the maximum used CC and HQ content was 2.0 wt% during the WBPU dispersions. The CC and HQ attachments in PU were theoretically analyzed by DFT calculation. All synthesized dispersions used an adhesive material to bond nylon fabrics. The adhesive strength was evaluated at a moderately high temperature. The hydrophobicity and thermal stability properties were also evaluated.

## 2. Materials and Methods

### 2.1. Materials

The base monomers were collected from Sigma Aldrich. The monomers such as 4, 4-dicyclohexylmethane diisocyanate (H_12_MDI), triethylamine (TEA), and ethylene diamine (EDA) were used after dehydration with 4 Å molecular sieves for seven days. Catechol (CC), hydroquinone (HQ), dimethylolpropionic acid (DMPA), Methyl ethyl ketone (MEK), N-Methyl-2-pyrrolidone (NMP) and dibutyltindilaurate were used as received.

### 2.2. WBPU Dispersion Preparation

The formulation of all coatings is summarized in Table 1. All respective dispersions were prepared by pre-polymer process mainly following our previous reports [10,15]. All the reactions were carried out in a nitrogen atmosphere. The calculated amount of PTMG was degassed before charging the other monomers. DMPA/NMP (10 g) solution was added to the polyol for 30 min at 65 °C. Later the H_12_MDI was added to the reaction mixture in presence of the catalyst dibutyltindilaurate (0.01 wt%). Methyl ethyl ketone (5.0 wt%) was added to the reaction mixture to control the viscosity. The reaction was carried out for 3 h at 85 °C. Later the neutralization (added TEA), dispersion (H_2_O) and chain extension (EDA mixed with H_2_O) were carried out at 65 °C (30 min), 25 °C (30 min), and 40 °C (2 h), respectively. The CC or HQ (mixed with MEK, 1.0 wt%) was added to the dispersion and mixed for 30 min at the last step. Dispersed solid content were found at 29.0–30.5 wt%.

### 2.3. Preparation of WBPU Films

A Teflon disk of 7 cm diameter was used to make the respective film. About 12 g of each WBPU dispersion was poured onto that disk. The dispersion was dried at room temperature for 48 h. The dried films were further vacuum dried in an oven for 12 h at 50 °C.

### 2.4. Bonding of Nylon Fabrics

The prepared dispersions were further mixed with hardener (ARF 30, 0.50 wt%). The mixture was stirred for 10 min at room temperature. This mixture was used to sandwich between two nylon fabrics and hot-pressed at 100 °C and pressure 15 kN/m^2^. The condition was optimized based on our previous report [10].

### 2.5. Characterization

The typical functional groups of WBPU were identified by FT-IR (Impact 400D, Nicolet, 32 scans at a resolution of 4 cm^−1^) analysis. The hydrogen bond of films was also analyzed by FT-IR analysis. The melting temperatures (T_m_) of films were found by differential scanning calorimeter (DSC; model 220C, Seiko, Chibas, Japan) analysis. About 4.0 mg of WBPU film was used at the nitrogen gas atmosphere. The heating rate was fixed at 10 °C/min. The thermal gravimetry analysis (TGA) was performed in a Pyris 6 TGA (Perkim Elmer, IL, USA). The heating was from 30 to 500 °C at a heating rate of 10 °C/min. The adhesion property was measured with the United Data System tension meter according to the ASTM D 1876-01 (the peel resistance of adhesives, i.e., the T-peel test).

**Figure 1:**
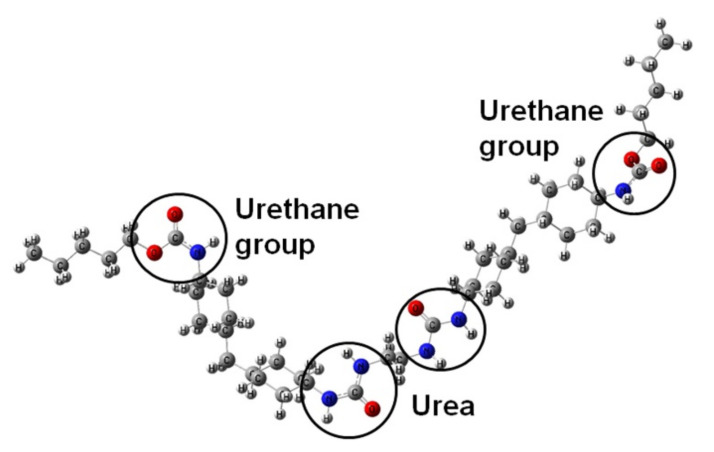
Optimized geometry of WBPU. Grey, red, blue, and white color circles represent carbon, oxygen, nitrogen, and hydrogen atoms, respectively.

DFT study: Water-borne polyurethane (WBPU) polymer is composed of urethane and urea linkage. Herein, two urethane groups linked with two urea groups were considered as a WBPU unit (Figure 1). To study the interaction between dihydroxybenze and WBPU, two isomers of dihydroxybenze (catechol and hydroxyquinone) have been considered. The initial geometries of WBPU, catechol, and hydroxyquinone are optimized using DFT methods, Becke three-parameter exchange functional^1^, and the Lee–Yang–Parr functional^2^ (B3LYP). The 6-31 + G (d, p) has been considered as a basis set. B3LYP method was found good for structure optimization, binding energies, Mulliken charges, dipole moments and IR spectra in 4,4′-diphenylmethane diisocyanate (MDI)-based polyurethane [19]. Two active sites, urethane and urea groups, have been considered in WBPU (Figure 1) for H-bond formation with catechol and hydroxyquinone. To compute complex formation binding energy Equation (1) has been used:

ΔE_BE,cp_ = E_WBPU-X_ − E_EBPU_ − E_X_ + δ_WBPU-X,BSSE_ (1)

where ΔE_BE,cp_ is the counterpoise corrected binding energy between WBPU and analyte. E_WBPU-X_, E_WBPU_ and E_X_ are the total energies of WBPU-X complex (X = catechol or hydroquinone), WBPU, and X, respectively. When the binding energy between two molecules approaching one another is calculated with the use of finite basis set, basis set superposition errors (BSSE) occur. Counterpoise method corrects this energy as shown in Equation (1). The real ground states for all optimized geometries are confirmed by the absence of imaginary frequencies corresponding to local minima on the potential energy surfaces.

Electronic parameters such as natural bond orbital (NBO) analysis with an NBO charge have been calculated with the above-mentioned level of theory. Time-dependent density functional theory (TD-DFT), TD-B3LYP/6-31 + G (d, p) has been used for excited-state optimizations of WBPU and WBPU-X complexes. The UV-visible spectra were plotted using the excitation energy and oscillator strength of the molecules in TDDFT calculations. All calculations presented herein are executed with the Gaussian16 program [20].

## 3. Results and Discussion

**Figure 2:**
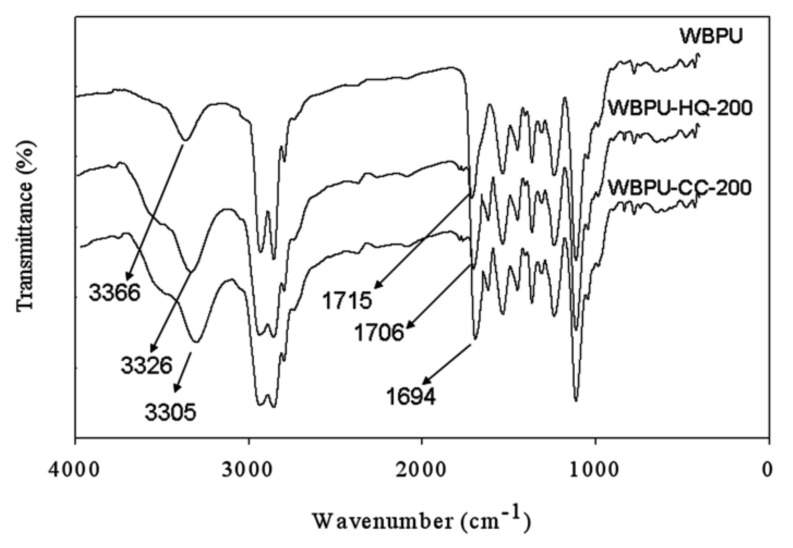
FT-IR spectra of WBPU.

All dispersions were prepared by an in-situ polymerization method following our previous report [10]. WBPU polymer was identified by FT-IR spectroscopy. Typical FT-IR spectra is given in Figure 2. Almost a similar type of spectra and pattern were recorded for all the polymers. A few typical peaks at 2919 (-C-H), 1624 (-C=C), 1247 (-C-N), 1144 (-C-O-C), 1001 (-C=C) cm^−1^ appeared almost at the same position for all the films. The peaks at 3430 and 1710 cm^−1^ imply NH and CO groups, respectively. The peaks of NH and CO groups, which are identical for urethane and urea confirmed PU polymer was prepared properly [10]. The peak at 3430 cm^−1^ was broadened after DHB addition. The wide peak is attributed to the presence of OH groups which appeared for DHB. The observation that both peaks at 3430 and 1710 cm^−1^ shifted to lower values with addition of DHB also implied hydrogen bonding by the addition of DHB. A much lower shifted value with higher DHB content was also recorded (not shown), which confirmed a higher level of HB. The large number of hydrogen bonding can be ascribed by available OH groups, which can easily participate in this bonding. Surprisingly, it was also found that a much lower shifting value was recorded for CC than HQ at the same composition. The difference is clearly seen in higher contents of CC and HQ (not shown). This means that CC involved more HB. The -OH position in CC might make a difference and have greater hydrogen bonding in WBPU.

**Figure 3:**
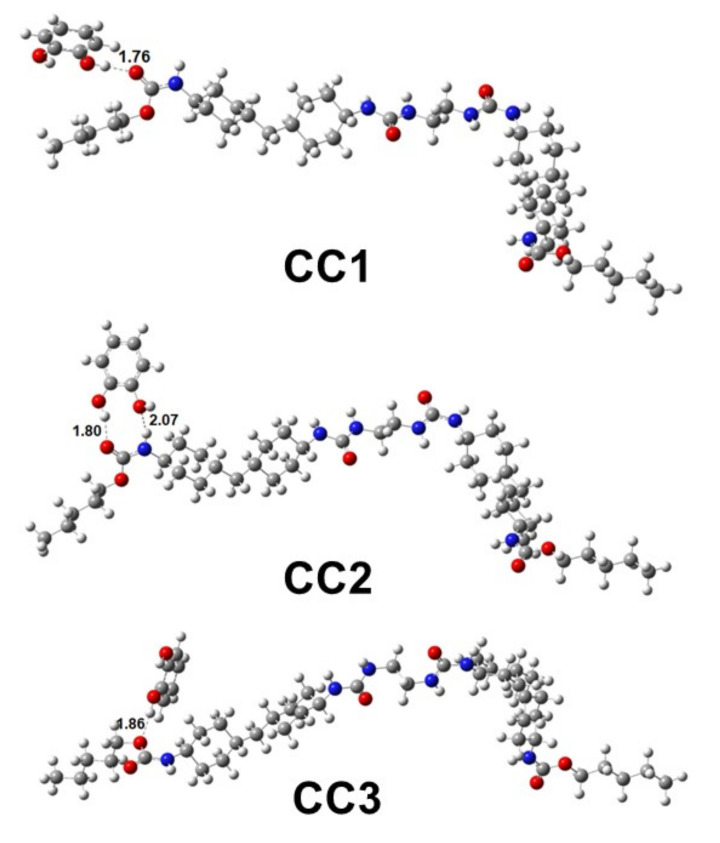
Three different approaches through which catechol binds with the urethane group of WBPU. Catechol forms hydrogen bonds to bind with WBPU. In CC1, CC2 and CC3 approaches, CC interacts with oxygen atom of carbonyl group, oxygen atom of carbonyl and nitrogen of amine, and oxygen atom of ether group of urethanes in WBPU, respectively.

**Figure 4:**
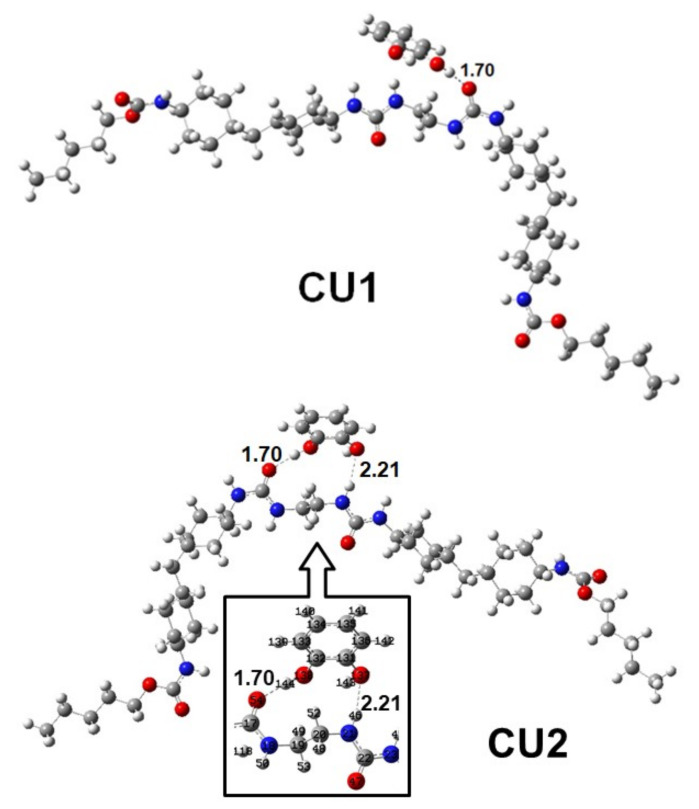
Catechol binds with urea group of WBPU through two approaches. In the CU1 approach, catechol forms one hydrogen bond with the carbonyl group of urea and in CU2, it forms two hydrogen bonds with two urea groups. The inside figure is a close view of the interaction between catechol and WBPU with atom levels.

To confirm the hydrogen bonding of CC and HQ in WBPU, the DFT method was employed. In WBPU (see Figure 1), urethane and urea groups are two active sites where analytes (catechol and hydroquinone) can bind. Firstly, a catechol has been placed 3.0 Å above the urethane group in WBPU and optimized with the above-mentioned level of theory. The three different approaches (see Figure 3) have been discussed below for WBPU-catechol complex formation through H-bonds. In the first approach (CC1), the –OH_catechol_ forms a H-bond with the oxygen atom of the carbonyl group in urethane with a bond length 1.76 Å. In the second approach (CC2), two –OH_catechol_ form two H-bonds with oxygen atom and nitrogen atom of carbonyl and amine group in urethane with 1.80 Å and 2.07 Å bond lengths, respectively. In the third approach (CC3), one –OH_catechol_ forms a hydrogen bond with the oxygen atom of ether group in urethane with 1.86 Å bond length. Among the three approaches, CC1 exhibits the most significant complex formation energy (ΔE_BE,cp_) −8.81 kcal/mol between WBPU and catechol. In the CC2 and CC3 approaches, the energies are −6.23 kcal/mol and −3.66 kcal/mol, which are 2.42 kcal/mol and 4.75 kcal/mol less than that of CC1, respectively. Later on, the catechol is placed on urea linkage and forms H- bonds through two approaches (CU1 and CU2) (Figure 4). In CU1, one -OH group of catechol forms a H-bond with the oxygen atom of urea with bond length 1.70 Å. Here, the calculated *ΔE_BE_* is −11.84 kcal/mol, which is 3.41 kcal/mol higher than in the CC1 approach. In CU2, two –OH groups form two H-bonds with two linked urea groups. One -OH group forms an H-bond with the oxygen atom of one urea with bond length 1.67 Å and another –OH group forms H-bond with the hydrogen atom of –NH group of urea with bond length 2.21 Å. In the CU2 approach, the calculated binding energy is −12.36 kcal/mol, which is 0.52 kcal/mol higher than the CU1 approach.

**Figure 5:**
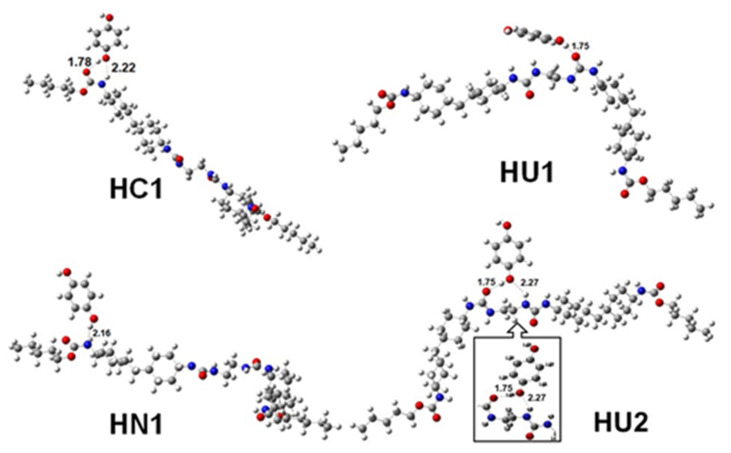
Hydroquinone binds with WBPU through four approaches. Two approaches (HC1 and HN1) for binding with the urethane group and two approaches (HU1 and HU2) to bind with the urea group in WBPU. The inside figure is a close view of the interaction between hydroquinone and WBPU with atom levels.

Similarly, hydroquinone was placed on the urethane group at a 3.0 Å distance. Here, hydroquinone followed two approaches (HC1 and HN1) to interact with the urethane group (Figure 5). In the HC1 approach, -OH_hydroquinone_ forms two H-bonds with urethane, one with the oxygen atom of carbonyl group and another with the hydrogen atom of -NH group in urethane with bond lengths 1.78 Å and 2.22 Å, respectively. The calculated *ΔE_BE_* of this approach is −9.56 kcal/mol, which is 2.80 kcal/mol lower than the CU2 approach of catechol. In the HN1 approach, hydrogen atom of -OH_hydroquinone_ forms a hydrogen bond with the nitrogen atom of –NH in the urethane group with a bond length 2.16 Å. Here, *ΔE_BE_* is 6.66 kcal/mol lower than the HC1 approach. When hydroquinone is placed over urea groups, HU1 and HU2, two approaches are observed (Figure 5). In HU1, -OH_hydroquinone_ forms H-bond with oxygen atom of carbonyl group of urea with bond length 1.75 Å. Here, the calculated *ΔE_BE_* is −10.29 kcal/mol which is 0.73 kcal/mol higher than that of the HC1 approach. Nevertheless, it is 1.22 kcal/mol lower than that of the CU2 approach. In the HU2 approach, -OH_hydroquinone_ forms two hydrogen bonds with two ureas, one with a carbonyl group of one urea and another with the –NH group of another urea with bond lengths 1.75 Å and 2.27 Å, respectively. The highest complex formation energy, −11.51 kcal/mol, has been obtained in this approach. This value is 1.10 kcal/mol lower than that of the CU2 approach. It implies that catechol can bind with WBPU through a CU2 approach more strongly than hydroquinone. All complex formation energies are shown in Table 2.

The natural bond orbital (NBO) analysis has been performed to estimate the charge transfer by catechol and hydroquinone and to confirm the H-bond formation during complex formation with WBPU. In each approach, catechol and hydroquinone detract charges from WBPU, as shown in Table 2. In the CU2 approach, catechol detracts the most considerable amount of charge 0.038 e^−^ from WPU. On the other hand, in the HU2 approach, hydroquinone detracts 0.031 e^−^ the amount of charge from WBPU which is 0.007 e^−^ less than that of the CU2 approach. It manifests in a stronger binding between WBPU and catechol than that of WBPU and hydroquinone. Besides this, to analyze the interaction for intermolecular H-bond formation, NBO analysis has been considered. This technique can provide the most possible natural Lewis structure picture of orbitals. In the complex, an H-bond forms due to intermolecular interactions which arise from electron density transfer from filled lone pair electrons (*n*) of the “Lewis base” into the unfilled antibonding (σ*) of the “Lewis acid”. The strength of interactions, E^2^, is estimated by second-order perturbation theory [21]. In WBPU-catechole complex, NBO analysis evidence the formation of two H-bonds, of which one is n1(O54)→σ*(O138-H144) with the highest stabilization energy 11.68 kcal/mol and another is n2(O137)→σ*(N21-H46) with stabilization energy 0.80 kcal/mol. Between two H-bonds, the C-O---H-O bond is more stable than N-H---O-H. To find the effect of H-bond on the two nearest bonds C17-O54 in WBPU and O138-H144 in catechol, we have analyzed Wiberg bond index (WBI), which ensures the bond strength relative to the overlap population [4]. The WBIs of C17-O54 and O138-H144 in isolate WBPU and catechol are 1.5705 and 0.7118, respectively. After H-bond formation, the WBIs of C17-O54 and O138-H144 decrease to 1.4614 and 0.5934, respectively. A new H-bond formation between oxygen and hydrogen of O54 and H144 reduces the strength of the two bonds. Similarly, for the second H-bond between H46 and O137, the WBIs of N21-H46 and O137-H148 decrease from 0.7881 and 0.6995 to 0.7589 and 0.5934, respectively.

In the WBPU-hydroquinone complex, NBO analysis evidences the formation of two H-bonds, C17-O54—H144-O140 and N21-H46—O140-H144. The H-bond C17-O54—H144-O140, is observed through n2(O54)→σ*(O140-H144) charge transfer with the highest stabilization energy of 12.16 kcal/mol. Another H-bond N21-H46—O140-H144 is formed through n1(O140)→σ*(N21-H46) charge transfer with stabilization energy 3.53 kcal/mol. Between the two H-bonds, the C17-O54—H144-O140 bond is more stable than N21-H46---O140-H144 by 8.63 kcal/mol. The WBIs of O137-H143 and O140-H144 is 0.7275. After H-bond formation, the WBIs of C17-O54, N21-H46 and O140-H144 decrease to 1.4718, 0.7588 and 0.6231, respectively. Therefore, two newly formed H-bonds decrease the strength of the three bonds. However, in this case, the O137-H143 bond does not take part in any H-bond formation and its WBI does not change.

**Figure 6:**
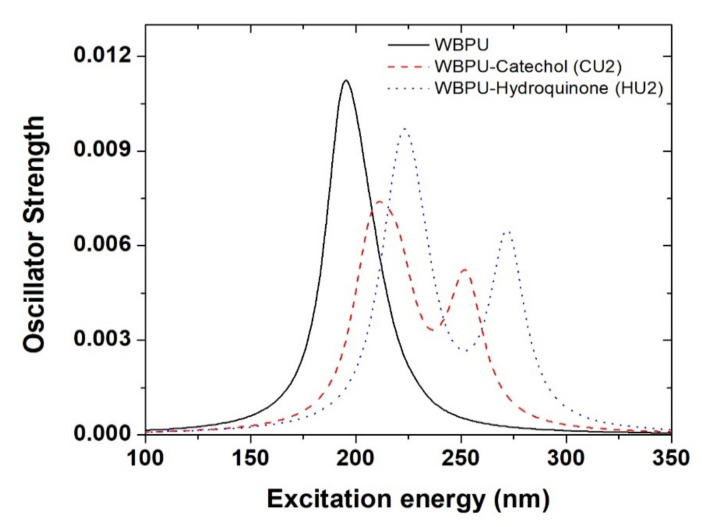
UV-vis spectra of isolated WBPU and WBPU-X complexes calculated at B3LYP/6-31 + G (d, p) level of theory. In the figure, solid, dashed, and dotted lines represent the UV-vis spectra of isolated WBPU, WBPU-catechol, and WBPU-Hydroquinone complexes, respectively.

For further evidence of the formation of WBPU-catechol and WBPU-hydroquinone complexes through H-bonds, we calculated UV-vis spectra of isolated WBPU and WBPU-X complexes using TD-DFT with the above-mentioned level of theory presented in Figure 6. The estimated *λ_max_* of isolated WBPU is found at 195 nm, which corresponds to the π→π* transition. Two WBPU-X complexes found through CU2 and HU2 approaches were considered for UV-vis spectra calculation because the largest complex formation binding energies for WBPU-catechol and WBPU-hydroquinone have been obtained through these two approaches. For the WBPU-catechol complex, the *λ_max_* is found at 211 nm with a weak band at 252 nm. For WBPU-hydroquinone, the *λ_max_* appears at 223 nm with a weak band at 272 nm. In both WBPU-catechol and WBPU-hydroquinone complexes, the *λ_max_* are red-shifted by 16 nm and 28 nm compared to isolated WBPU. This redshifting of *λ_max_* and newly appeared weak bands confirm the formation of WBPU-catechol and WBPU-hydroquinone complexes.

Conventional WBPU coatings are mainly hydrophilic [10]. As the WBPU-CC materials have enriched hydrogen bonds, this polymer surface might have less interaction with water or moisture. At the same time, the WBPU-HQ samples might have more interactions. The overall interaction is usually reflected in their hydrophilicity/hydrophobicity. A greater interaction is reflected by showing their hydrophilicity, or conversely showing their hydrophobicity. The hydrophilicity/hydrophobicity was checked by the water contact angle test (see Table 3). The value increased with increasing CC and HQ content. In WBPU-CC films, the initial value was at 68° (0.5 wt% CC), which increased to 82° with 2.0 wt% CC content. Meanwhile, the WBPU-HQ films also showed an almost similar extent of increase (changed from 67° to 74°). Although the value increased in both cases, the CC films showed higher values with the same CC or HQ content. This indicates a different dynamic of surface in the presence of CC or HQ due to the hydrogen bond. This also clearly implies the occurrence of orientation/dynamics of surface groups with CC and HQ content. Interestingly, WBPU-CC displayed hydrophobic properties, whereas WBPU-HQ exhibited less hydrophobicity. This is attributed to the increased H-bonding in WBPU-CC, which may not allow polar water molecules to adhere to the surface. The more exposed H-bonding atoms in WBPU-HQ will lead to more interactions with water and thus reduce the contact angle. The maximum shifted value was found at 82°for WBPU-CC-200 (2.0 wt% CC).

**Figure 7:**
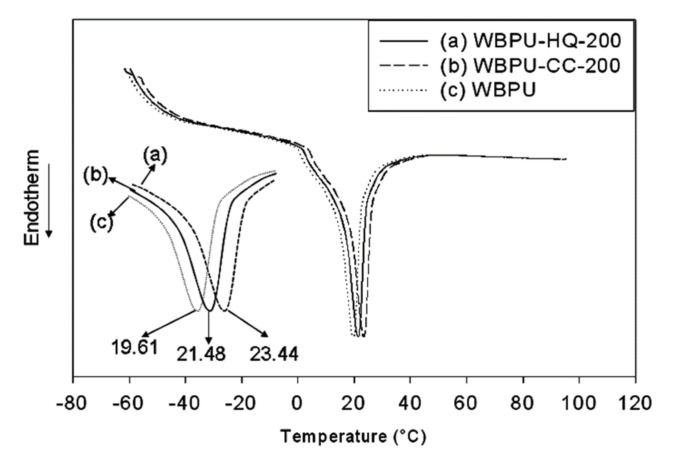
Typical DSC thermograms of WBPU.

The melting temperature (T_m_) of all films was evaluated by DSC. The T_m_ values and DSC curves are summarized in Table 3 and Figure 7. The T_m_ of WBPU, which was free from CC or HQ, appeared at 19.61 °C, whereas the T_m_ of WBPU-CC and WBPU-HQ films were recorded above 19.61 °C. The T_m_ shifted to higher values gradually with increasing either CC or HQ content. Usually, a clear higher shifting value of T_m_ is recorded when there is a huge difference in the hydrogen bonding of polymers [10]. This clearly indicates that the WBPU-CC and WBPU-HQ possess more hydrogen bonding with the addition of either CC or HQ. The difference was more prominent with higher CC/HQ content. With the addition of CC, the T_m_ shifted 0.50 °C and 3.83 °C with 0.50 wt% and 2.0 wt%, respectively. At the same time, comparing all the polymers between WBPU-CC and WBPU-HQ, the T_m_ shifting was always higher for WBPU-CC than WBPU-HQ with a fixed CC and HQ content. The maximum shifted values of 3.83 °C and 1.87 °C were recorded with 2.00 wt% CC and HQ addition, respectively. These findings lead to the conclusion that hydrogen bonding worked here as dictating factor and it helped to have materially different properties.

**Figure 8:**
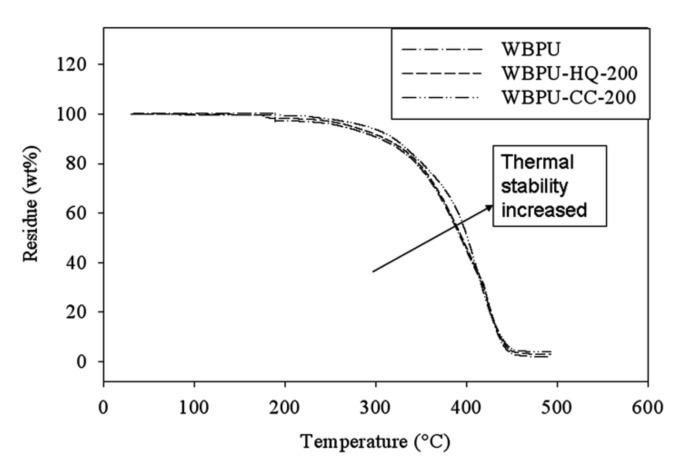
Typical TGA curves of WBPU.

The TGA was applied to evaluate the effect of hydrogen bonds on the thermal stabilities of films. The typical TGA thermographs are shown in Figure 8. All the graphs are showing similar trends, indicating that the addition of CC or HQ in WBPU has no interference with their degradation properties. It can clearly be seen from the graphs that all of the films showed two steps of degradation. The first step was slower degradation, and the second step was faster degradation. The degradation criteria confirm that the polymers are mainly stable up to their first degradation step. The degradation temperature is enhanced by the addition of either CC or HQ. It can also be seen that the degradation temperature is always higher for CC than for HG. This confirmed that the CC-based WBPU had a better thermal stability. This can be ascribed to hydrogen bonds. As the bond energies of CC-based films are higher, more energy is needed for degradation. Thus, the thermal stability automatically improved for CC-based films.

All the dispersions were used to bonded nylon fabrics. The WBPU materials act as an adhesive material. The adhesive strength was measured at room temperature and a defined moderate–high temperature at 80 °C. The respective adhesive strength values are summarized in Table 3. The adhesive strength increased with the addition of either CC or HQ. The adhesive strength continued to increase with the increase of CC or HQ content. However, the increasing rate was not the same with CC or HQ addition. As expected, the CC series showed a higher adhesive strength compared to those of the HQ series. The hydrogen bond, which was higher in the CC series, made the difference for higher adhesive strength. As hydrogen bond energy was higher in CC-based adhesive materials, the mechanical interlocking was stronger in CC-based samples. Obviously, more energy was needed to de-bond it. This was reflected in the higher adhesive strength. At a higher CC content, maximum energy was required to pull off and thus the maximum adhesive strength was recorded with 2.0 wt% CC content. The adhesive strength increased almost 25% after adding 2.0 wt% CC in WBPU adhesive material. The adhesive strength was also checked at moderately high temperatures. The adhesive strength dropped 72% of WBPU. However, both CC and HQ worked against dropping the adhesive strength. Both WBPU-CC and WBPU-HQ series showed higher adhesive strength than the pristine WBPU adhesive at 80 °C. The maximum resistance was found with higher CC or HQ content (2.0 wt%). From the adhesive strength values, it was also shown that the decrease rate was always higher for HQ than for CC. At 80 °C, the adhesive strength decreased 41% and 17% for HQ and CC, respectively. The hydrogen bond worked against the dropping and it kept strongly the mechanical encoring with nylon fabrics [10]. The hydrogen bond still worked strongly to oppose the dropping of adhesive strength at this temperature. The hydrogen bond energy was at a maximum in the WBPU-CC-200 sample (2.0 wt% CC), and thus the mechanical interlocking was strong enough even at that temperature; eventually, the adhesive strength was slightly affected at 80 °C for the WBPU-CC-200 sample.

## 4. Conclusions

The DHB isomer, as CC and HQ, with different -OH positions used in WBPU and the effect of structural isomer on HB, was investigated. By the DFT method, it confirmed that the CC rather than the HQ, can bind the urethane/urea group more strongly by a hydrogen bond. The FT-IR analysis of synthesized WBPU materials also matched with their theoretical result as a higher level of hydrogen bond was also found in WBPU-CC compared with the WBPU-HQ series. As expected, the thermal stability and adhesive strength values were also higher in the WBPU-CC series compared to the WBPU-HQ series. Significantly higher values were recorded at 2.00 wt% CC content in WBPU. The adhesive strength increased almost 25% at room temperature. The adhesive strength was 7.4 kN/m at 80 °C, which is compatible in many cases.

## Figures and Tables

**Table 1:** Sample designation and composition of coatings.

Coating	Composition (Mole)					CC (wt%)	HQ (wt%)
	PTMG	DMPA	TEA	EDA	H_12_MDI		
WBPU	1.46	1.84	1.84	0.70	4.00		…
WBPU-CC-50	1.46	1.84	1.84	0.70	4.00	0.50	…
WBPU-CC-100	1.46	1.84	1.84	0.70	4.00	1.00	…
WBPU-CC-150	1.46	1.84	1.84	0.70	4.00	1.50	…
WBPU-CC-200	1.46	1.84	1.84	0.70	4.00	2.00	…
WBPU-HQ-50	1.46	1.84	1.84	0.70	4.00	…	0.50
WBPU-HQ-100	1.46	1.84	1.84	0.70	4.00	…	1.00
WBPU-HQ-150	1.46	1.84	1.84	0.70	4.00	…	1.50
WBPU-HQ-200	1.46	1.84	1.84	0.70	4.00	…	2.00

**Table 2:** Complex formation binding energy (*ΔE_BE)_*), charge transferred by catechol (q_catechol_) and hydroquinone (q_hydroqinone_) after complex formation calculated at B3LYP/6-31 + G (d, p) level of theory.

Complex	Approach	*ΔE_BE_* (kcal/mol)	q_catechol_ (e^−^)	q_hydroquinone_ (e^−^)
WBPU-catechol	CC1	−8.81	−0.034	
	CC2	−6.23	−0.019	
	CC3	−3.66	−0.025	
	CU1	−11.84	−0.039	
	CU2	−12.36	−0.038	
WBPU-hydroquinone	HC1	−9.56		−0.032
	HN1	−2.90		−0.022
	HU1	−10.29		−0.030
	HU2	−11.51		−0.031

**Table 3:** The properties of WBPU coatings.

Sample	Water Contact Angle (nm)	Adhesive Strength (kN/m)		Tm
		25 °C	80 °C	
WBPU	67 ± 1	7.1 ± 0.1	2.0 ± 0.1	19.61
WBPU-CC-50	68 ± 1	7.3 ± 0.1	2.0 ± 0.2	19.65
WBPU-CC-100	71 ± 1	7.6 ± 0.1	2.8 ± 0.1	20.10
WBPU-CC-150	74 ± 1	8.1 ± 0.2	4.4 ± 0.2	21.86
WBPU-CC-200	82 ± 1	8.9 ± 0.1	7.4 ± 0.1	23.44
WBPU-HQ-50	67 ± 2	7.2 ± 0.1	2.1 ± 0.1	19.63
WBPU-HQ-100	69 ± 1	7.3 ± 0.1	2.6 ± 0.1	19.73
WBPU-HQ-150	72 ± 2	7.7 ± 0.1	3.5 ± 0.2	20.50
WBPU-HQ-200	74 ± 1	8.0 ± 0.1	4.7 ± 0.3	21.48

## Data Availability

Not applicable.
